# A Case of Success: Complete Response to Radium-223 in Metastatic Castration-Resistant Prostate Cancer

**DOI:** 10.7759/cureus.53637

**Published:** 2024-02-05

**Authors:** Inês Soares de Pinho, Miguel Esperança-Martins, Bárbara Machado, Sara Dâmaso, Raquel Lopes Brás, Guilhermina Cantinho, Isabel Fernandes, Luís Costa

**Affiliations:** 1 Medical Oncology, Hospital de Santa Maria, Centro Hospitalar Universitário Lisboa Norte (North Lisbon University Hospital Center), Lisbon, PRT; 2 Oncology, Luis Costa Lab, Instituto de Medicina Molecular (Institute of Molecular Medicine), Lisbon, PRT; 3 Medical Oncology, Centro Hospitalar Entre Douro e Vouga (Hospital Center Entre Douro e Vouga), Santa Maria da Feira, PRT; 4 Nuclear Medicine, Faculdade de Medicina, Universidade de Lisboa (Faculty of Medicine, University of Lisbon), Lisbon, PRT; 5 Oncology, Hospital CUF Descobertas, Lisbon, PRT; 6 Research, Comprehensive Health Research Center, Nova Medical School, Lisbon, PRT; 7 Research, EpiDoC, Nova Medical School, Lisbon, PRT

**Keywords:** bone disease, response, flare, radium-223, prostate cancer

## Abstract

Radium-223 dichloride (Ra223) is the first targeted alpha agent approved for treating metastatic castration-resistant prostate cancer (mCRPC) with bone-exclusive disease. A benefit in overall survival and time to the first symptomatic skeletal-related event was shown in the Alpharadin in Symptomatic Prostate Cancer Patients (ALSYMPCA) trial. However, this trial did not describe a bone scan response to Ra223, and there is no universal consensus about how it should be monitored. Furthermore, a scintigraphy flare phenomenon may lead to false-positive tracer uptake in responsive cases, thereby misleading the interpretation of imaging results.

We present the case of a 67-year-old male with mCRPC and exclusive bone disease treated with Ra223. The bone scintigraphy after the end of the treatment showed an apparent aggravation of the lesions, corresponding to a flare phenomenon, with an almost complete resolution after three months. The patient maintained a scintigraphic response for seven months.

## Introduction

More than 90% of the patients with metastatic castration-resistant prostate cancer (mCRPC) develop bone metastasis and have an increased risk of skeletal-related events (SREs) that involve a myriad of complications, including pain (the most common symptom), pathologic fractures, and spinal cord compression, which occur in 20% to 50% of the patients. These complications most often require radiation therapy or surgery for bone metastases and are the major causes of morbidity, decreased quality of life, and increased treatment costs [1]. In mCRPC, unlike other solid tumors, bone disease and its complications are independently associated with increased mortality [2,3].

It is acknowledged that prostate cancer shows an exquisite tropism to metastasize in the bone, and bone-only disease is not uncommon. One underlying mechanism for this is the overactivation of the receptor activator of NF-kB ligand (RANKL), its receptor RANK, and the osteoprotegerin (OPG) signaling pathway. The RANK/RANKL/OPG plays a predominant role in the development of bone-only metastases through the activation of RANKL osteoclastic-mediated bone resorption with the release of matrix growth factors that can promote the growth of tumor cells and create a positive feedback loop [4]. This system can be targeted by the RANKL antagonist, denosumab. Bisphosphonates inhibit osteoclastic bone resorption by attaching to hydroxyapatite crystal binding sites, particularly in areas with active resorption. As osteoclasts resorb bone, the bisphosphonate embedded in the bone is released, which impairs the osteoclast’s ability to continue bone resorption. Bone-targeted agents (BTAs), like bisphosphonate zoledronic acid and denosumab, are approved drugs in the treatment scenario of mCRPC with bone metastases for the prevention of SREs [5].

The use of targeted radionuclides for the treatment of mCRPC serves different purposes. Radium-223 dichloride (Ra223) has shown survival benefits and is approved in later lines for the treatment of mCRPC. Strontium-89 and samarium-153 ethylenediamine tetramethylene phosphonate are valuable weapons for symptomatic control, delaying skeletal events, and relieving pain related to bone lesions [6-8]. 

Radium-223 is a bone-targeted alpha-emitting agent that acts as a calcium-mimetic radioisotope. It is selectively taken up into areas of newly formed bone stroma with increased metabolic activity and high cellular turnover, like those encircling bone metastases [9]. Once it reaches the bone, its decay allows the deposition of high-energy alpha particles with short-range (<100 μm) [10], inducing double-strand DNA breaks with cytotoxic effects on tumor cells while minimizing the toxic effects of the surrounding healthy tissues and limiting the bone marrow toxicity [11,12]. The Alpharadin in Symptomatic Prostate Cancer Patients (ALSYMPCA) trial [13], a phase III randomized double-blinded controlled study, evaluated the benefits of Ra223 vs. placebo in patients with mCRPC. All the patients had multiple bone metastases (two or more), no known visceral metastases, and had either progressed on docetaxel or were not candidates for docetaxel chemotherapy. The trial showed that Ra223 increased both median overall survival (14.9 months vs. 11.3 months, hazard ratio (HR) 0.70, 95% CI 0.58-0.83) and median time to the first symptomatic SRE (15.6 months vs. 9.8 months, HR 0.66, 95% CI 0.52-0.83), defined as the first use of external beam radiation therapy (EBRT) for symptom relief, new pathologic fracture, spinal cord compression, or tumor-related orthopedic surgery intervention. These results led to the approval of Ra223. In 2019, the phase III ERA-223 trial [14] demonstrated that the concomitant use of Ra223 plus abiraterone and prednisolone had particularly harmful effects. This combination resulted in increased mortality and a higher frequency of bone fractures compared to placebo and did not improve symptomatic skeletal event-free survival. These results led to the emission of a black box warning regarding the combined use of Ra223 and abiraterone plus prednisone by the FDA and European Medicines Agency (EMA).

We present the case of a 67-year-old male with mCRPC with exclusive bone involvement. He was treated in the third line with Ra223 after progressing under abiraterone and docetaxel. A flare phenomenon was first verified after the completion of the treatment, but a subsequent profound and durable response of almost all the bone lesions was verified.

## Case presentation

In this case report, we present the clinical journey of a 67-year-old man who underwent a radical prostatectomy in 2012, revealing a Gleason score of 7 (3+4), International Society of Urological Pathology (ISUP) grade 2, pT3b pN1, with positive margins (R1). Following surgery, the patient experienced a nadir prostate-specific antigen (PSAt) of 0.47 ng/mL after five months and subsequently received external beam radiotherapy (RTE) directed to the prostate bed (65 Gy), achieving a PSAt nadir of 0.16 ng/mL.

The emergence of biochemical recurrence in 2015 prompted a prostate-specific membrane antigen (PSMA)-positron emission tomography (PET) scan, unveiling a singular bone lesion in a rib and the involvement of a lone lymph node in pre-sacral topography. In the context of low-volume metastatic castration-sensitive prostate cancer (mCSPC), androgen deprivation therapy (ADT) was initiated with leuprorelin (45 mg every six months), resulting in decreasing PSAt values and a rapid decline in testosterone to castration levels. Documented biochemical progression and castration resistance in 2017 led to the initiation of abiraterone (1000 mg/day) and prednisolone (10 mg/day), demonstrating consistent undetectable PSAt levels and no additional lesions in periodic PET-PSMA evaluations until 2019.

In February 2019, a biochemical progression ensued, revealing new bone lesions (<3) in a PET-PSMA scan. Consequently, the patient was prescribed docetaxel (75 mg/m2 every three weeks) and denosumab (120 mg/month). Progression in PET-PSMA was documented in August 2021 and prompted the proposal of six cycles of Ra223 (every four weeks) completed in January 2022 without dose delays. The patient had mild adverse effects from the treatment, such as moderate asthenia and occasional diarrhea, which never led to treatment being interrupted or postponed.

**Figure 1:**
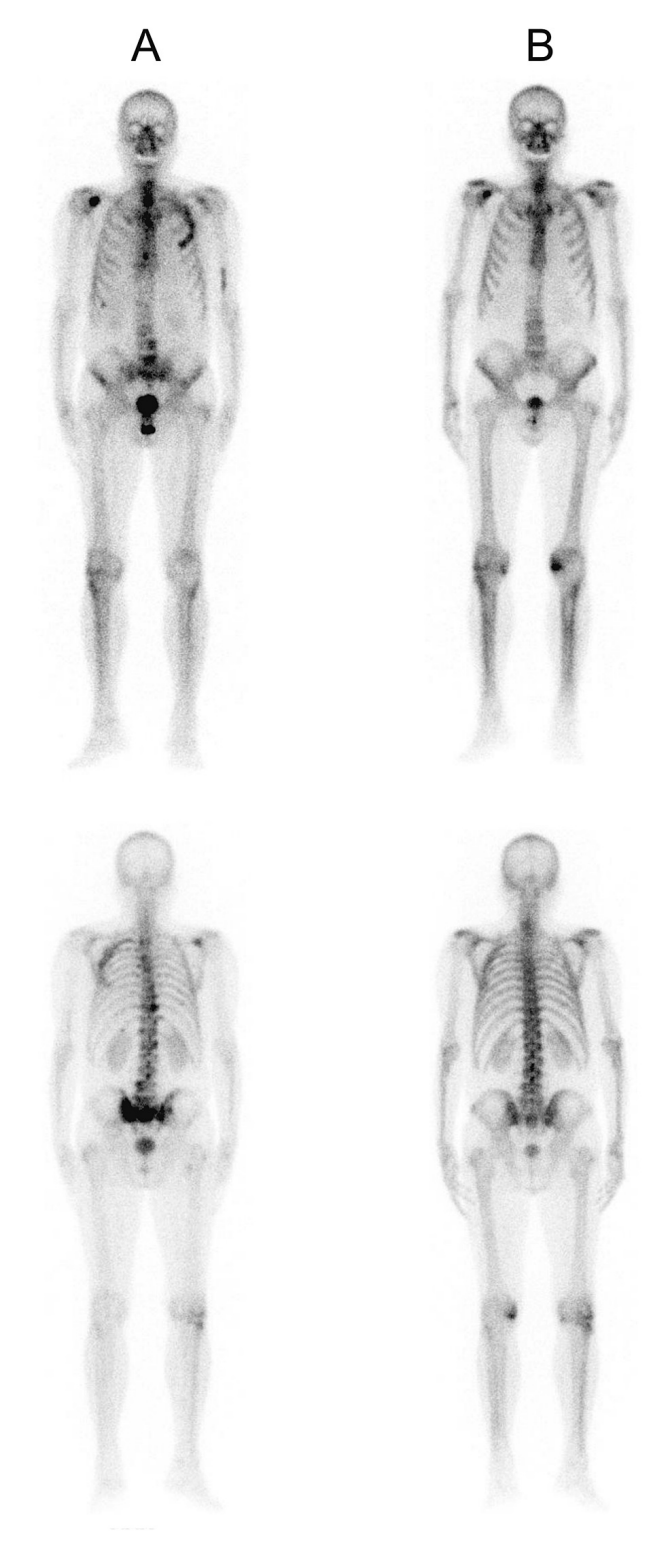
Comparison of the bone scintigraphy A: The bone scan conducted one month after Ra223 cycles, reveals multiple osteoblastic lesions at the distal sternal bone, dorsal, and lumbar vertebral bodies, at the third left costal arch, right glenohumeral joint, and pelvis. B: The bone scan done three months post-treatment, shows only a slight increase in uptake at the third left costal arch and in the glenohumeral joint. The lesions here show much less metabolic activity than in the previous bone scan (A). Ra223: Radium-223 dichloride

Remarkably, PSAt values exhibited a progressive increase during the Ra223 treatment cycles, reaching 179 ng/mL and maintaining a similar trend in the first evaluation post-treatment (223 ng/mL) (Tables 1-2). A bone scintigraphy one month after Ra223 cycles indicated an exacerbation of bone disease compared to the baseline scan (Figure 1). The subsequent evaluation, three months post-treatment, revealed a PSAt value of 0.34 ng/mL, and new bone scintigraphy displayed a nearly complete disappearance of all bone lesions (Figure 1). Continued close follow-up revealed an asymptomatic patient with consistently undetectable PSAt levels and no new bone or visceral lesions in imaging studies for seven months after that.

**Table 1:** The PSAt and ALP values one month and three months after after Ra223 treatment The PSAt levels dramatically dropped after the initial rise, whilst ALP levels steadily decreased. PSAt: Prostate-specific antigen; ALP: Alkaline phosphatase; Ra223: Radium-223 dichloride

Parameters	Values one month after Ra223 treatment	Values three months after Ra223 treatment
PSAt (ng/ml)	213-233	0.7-0.34
ALP (U/L)	68-60	60-54

**Table 2:** Evolution of the PSAt levels during the treatment with Ra223 and the four months after the treatment PSAt: Prostate-specific antigen; Ra223: Radium-223 dichloride

During Ra223 (cycles)	1	2	3	4	5	6	After Ra223 (months)	1	2	3	4
PSAt (ng/ml)	73.2	82.1	112	123	146	179	PSA (ng/ml)	223	6.1	0.76	0.34

## Discussion

Radium-223, an alpha-emitting radiopharmaceutical, has demonstrated efficacy in improving overall survival and delaying symptomatic skeletal events in patients with mCRPC, particularly those with bone metastases. It selectively targets areas of increased bone turnover, such as bone metastases, delivering targeted alpha radiation. The ALSYMPCA trial provided pivotal evidence for Ra223's efficacy. This phase III trial demonstrated a significant improvement in overall survival in patients receiving Ra223 compared to placebo, emphasizing its role as a valuable therapeutic option in mCRPC [13].

The case's intriguing aspect is the observed rise in PSAt levels during and after Ra223 treatment, followed by a subsequent decline and almost complete disappearance of bone lesions. This fluctuation in PSAt levels underscores the complexity of response assessment in patients undergoing Ra223 therapy. Traditional response criteria, based on PSAt kinetics, may not fully capture the nuanced effects of Ra223 on bone lesions.

There are limitations to PSAt as a surrogate marker. As we saw in this clinical case, a delayed PSAt response can occur. Radium-223's impact on bone lesions may precede changes in PSAt levels, leading to a delayed PSAt response. This temporal discordance challenges the conventional use of PSAt as a real-time marker of treatment efficacy. 

Additionally, imaging modalities like bone scintigraphy, while valuable, may have limitations in assessing response due to the heterogeneity of bone lesions and potential flare phenomena, where an initial increase in lesion visibility occurs before subsequent improvement. This phenomenon called flare is an osteoblastic bone healing reaction (callus formation) that can cause an initial rise in radiotracer uptake, with an increased number or size of lesions or increased metabolic activity in bone scintigraphy, and subsequent stabilization or improvement after three to six months [15,16], when the production of new bone slows down and, consequently, the radiotracer uptake falls. The flare can be accompanied by transient pain and/or transient PSAt increase [17]. Notwithstanding, the biological mechanisms underlying this phenomenon are still elusive and require further clarification. Flare is estimated to occur in about 10% of patients undergoing radiometabolic therapy for bone metastasis, using both radiopharmaceuticals based on bone-seeking radionucleotides labeled with beta-emitting isotopes, such as 153samarium or 186rhenium, osteomimetic agents, such as strontium-89 [16], and alpha-emitting agents, such as Ra223.

In the ALSYMPCA trial, imaging for monitoring response to Ra223 was not mandatory. Thus, there is a lack of information and uncertainty concerning the interpretation of bone scintigraphy acquired during and after treatment with Ra223 [15]. To mitigate this diagnostic challenge, Prostate Cancer Working Group 3 guidelines [18] recommend controlling the flare using a 2+2 rule, where the presence of two new bone lesions on the next scan is needed to confirm bone progression identified on the first scan performed during or after treatment.

This patient repeated bone scintigraphy two months after the first one (three months after the end of the Ra223 treatment). The new bone scintigraphy showed almost complete resolution of all the lesions related to the flare phenomenon. At this point, the PSAt value was 0.34 ng/ml.

The observed aggravation in bone disease during treatment and the subsequent improvement raise questions about the mechanisms underlying these fluctuations. Further research is warranted to elucidate the intricacies of Ra223's effects on bone microenvironments and how they manifest in imaging and PSAt dynamics. Exploring alternative biomarkers reflecting bone turnover and disease burden is essential to enhancing response evaluation accuracy. Additionally, advancements in imaging techniques, such as advanced PET tracers, such as Ga-68 PSMA-11 and Ga-68 PSMA-617, and functional imaging, may provide a more comprehensive understanding of treatment responses to these agents.

## Conclusions

The presented case underscores the need for a nuanced approach to evaluating therapeutic responses, especially in the context of evolving treatments like Ra223. One limitation of our work is the impossibility of measuring tumor volume using a single-photon emission computerized tomography (SPECT) bone scan, which would strengthen our conclusions. The limitations of traditional markers necessitate a multidimensional assessment, combining PSAt kinetics with advanced imaging and potentially exploring emerging biomarkers for a more comprehensive understanding of treatment response in mCRPC.
